# Deleting fibroblast growth factor 2 in macrophages aggravates septic acute lung injury by increasing M1 polarization and inflammatory cytokine secretion

**DOI:** 10.1186/s43556-024-00203-0

**Published:** 2024-10-22

**Authors:** Lingxian Yi, Yu Chen, Yaoyang Zhang, Haiquan Huang, Jiahui Li, Yirui Qu, Tujun Weng, Jiake Chai

**Affiliations:** 1 Department of Emergency, the Ninth Medical Centre of Chinese PLA General Hospital；Chinese PLA Medical School, Beijing, 100101 PR China; 2 The Fourth Medical Centre of Chinese PLA General Hospital; Chinese PLA Medical School, Beijing, 100048 PR China; 3 Senior Department of Orthopaedics, the Fourth Medical Centre, Chinese PLA General Hospital, Beijing, 100048 PR China

**Keywords:** Fibroblast growth factor 2, M1 macrophage, Sepsis, Acute lung injury, Inflammation

## Abstract

Septic lung injury is strongly associated with polarization of M1 macrophages and excessive cytokine release. Fibroblast growth factor (FGF) signaling plays a role in both processes. However, the impact of FGF2 deficiency on macrophage polarization and septic acute lung injury remains unclear. To investigate this, we obtained macrophages from FGF2 knockout mice and examined their polarization and inflammatory cytokine expression. We also eliminated endogenous macrophages using clodronate liposomes and administered FGF2 knockout or WT macrophages intravenously in conjunction with cecal ligation and puncture (CLP) surgery to induce sepsis. In vitro analysis by flow cytometry and real-time PCR analysis demonstrated that FGF2 deficiency resulted in increased expression of M1 markers (iNOS and CD86) and inflammatory cytokines (CXCL1, IL1β, and IL6), especially after LPS stimulation. Additionally, immunofluorescence demonstrated increased nuclear translocation of p65 NF-κB in FGF2 knockout macrophages and RNA-seq analysis showed enrichment of differentially expressed genes in the IL17 and TNFα inflammatory signaling pathways. Furthermore, in vivo experiments revealed that depletion of FGF2 in macrophages worsened sepsis-induced lung inflammation, lung vascular leak, and lung histological injury, accompanied by an increase in CD86-positive cells and apoptosis. Our study suggests that FGF2 deficiency in macrophages plays a critical role in the pathogenesis of septic ALI, possibly because of the enhanced M1 macrophage polarization and production of proinflammatory cytokines. These findings provide empirical evidence for potential therapeutic interventions targeting FGF2 signaling to modulate the polarization of M1 and M2 macrophages in the management of sepsis-induced acute lung injury.

**Supplementary Information:**

The online version contains supplementary material available at 10.1186/s43556-024-00203-0.

## Introduction

Sepsis is a life-threatening organ dysfunction caused by dysregulated host response to infection and is a major cause of admission and death in the intensive care unit (ICU) [1–3]. The Institute for Health Metrics and Evaluation (IHME) reported 48.9 million sepsis cases and 11 million sepsis-related deaths globally in 2017 [4]. Sepsis imposes a large clinical, social, and economic burden worldwide. Acute lung injury (ALI)/acute respiratory distress syndrome (ARDS) is a severe complication of sepsis, with a high mortality rate of 35–55% despite advanced intensive care support [5]. ALI is distinguished by a severe and acute inflammatory pulmonary impairment, characterized by heightened neutrophilic infiltration, alveolar edema induced by inflammatory neutrophil and macrophage migration into the alveolar epithelium, and release of multiple inflammatory mediators [6, 7]. Additionally, a significant number of proinflammatory cytokines, including tumor necrosis factor α (TNFα), interleukin 6 (IL6), and interleukin 1β (IL1β), can further exacerbate lung injury.

Recent research has indicated that macrophages play a crucial role in the pathogenesis of sepsis [8]. Macrophage activation and the ensuing inflammatory response mediated by macrophages are pivotal factors in the pathogenesis and progression of sepsis-induced ALI [9]. There are two distinct macrophage activation states: classically activated M1 macrophages and alternatively activated M2 macrophages. Upon exposure to lipopolysaccharide (LPS) or TNFα, macrophages undergo polarization toward the M1 phenotype. M1 macrophages exhibit proinflammatory properties and enhance host defense mechanisms by secreting proinflammatory mediators, such as cytokines, chemokines, and reactive oxygen/nitrogen species, to combat pathogens. In contrast, the polarization of macrophages into the M2 phenotype primed by IL4 has been shown to suppress immune responses, which are characterized by the expression of CD206, Ym1, and Arg1. Although macrophage-mediated polarization and inflammation are important factors in ALI pathogenesis, the precise molecular mechanisms underlying macrophage activation remain unclear.

Fibroblast growth factor (FGF) 2, also known as basic FGF, is a monomeric polypeptide comprising 146 amino acids that typically functions as a mitogen by interacting with the FGF receptors (FGFRs). Recent studies have demonstrated the potential therapeutic efficacy of FGF2 for the treatment of diverse medical conditions. Specifically, research has shown that FGF2 can reduce capillary permeability and inflammation in sepsis and improve coagulation abnormalities associated with the disease [10, 11]. Moreover, FGF2 has been implicated in immunomodulation in asthma and chronic obstructive pulmonary disease, where it plays a crucial role in regulating inflammatory cells and mediating interactions between immune and airway structural cells [12]. Additionally, a recent study demonstrates that the elimination of the low-molecular-weight FGF2 isoform in mice results in a shift in macrophage polarization toward an inflammatory (M1) phenotype [13]. FGF2 translation produces two protein isoforms from a single transcript: low-molecular-weight (LMW) and high-molecular-weight (HMW). The LMW isoform is cytoplasmic and is secreted, whereas the HMW isoform is typically found in the nucleus. The functional differences between the LMW and HMW isoforms of FGF2 present challenges in accurately predicting the effects of complete elimination of all FGF2 isoforms on macrophage behavior. The results obtained from the macrophage-specific knockout FGFR1 mouse model indicate that the absence of FGFR1 in macrophages may confer a protective effect in mice fed a high-fat diet by attenuating inflammatory responses [14]. Conversely, the lack of FGFR3 in macrophages leads to increased macrophage chemotaxis via activation of the NF-κB pathway, which ultimately exacerbates joint damage in mice [15]. However, the precise mechanism by which FGF2 signaling modulates macrophage polarization under inflammatory conditions and septic ALI remains unclear.

In this study, bone marrow-derived macrophages (BMDM) from FGF2 knockout (KO) mice were used to investigate macrophage polarization, inflammatory cytokine expression, and the effect of FGF2 deletion on molecular expression profiles. Additionally, the influence of FGF2 deletion in macrophages on sepsis was examined by depleting endogenous macrophages and reconstructing with FGF2 KO macrophages in a septic mouse model induced by cecal perforation ligation (CLP) to assess the impact and molecular mechanisms of FGF2 KO macrophages in septic ALI.

## Results

### FGF2 deficiency in macrophages increases their susceptibility to apoptosis and promotes a shift toward M1 macrophages

**Fig. 1:**
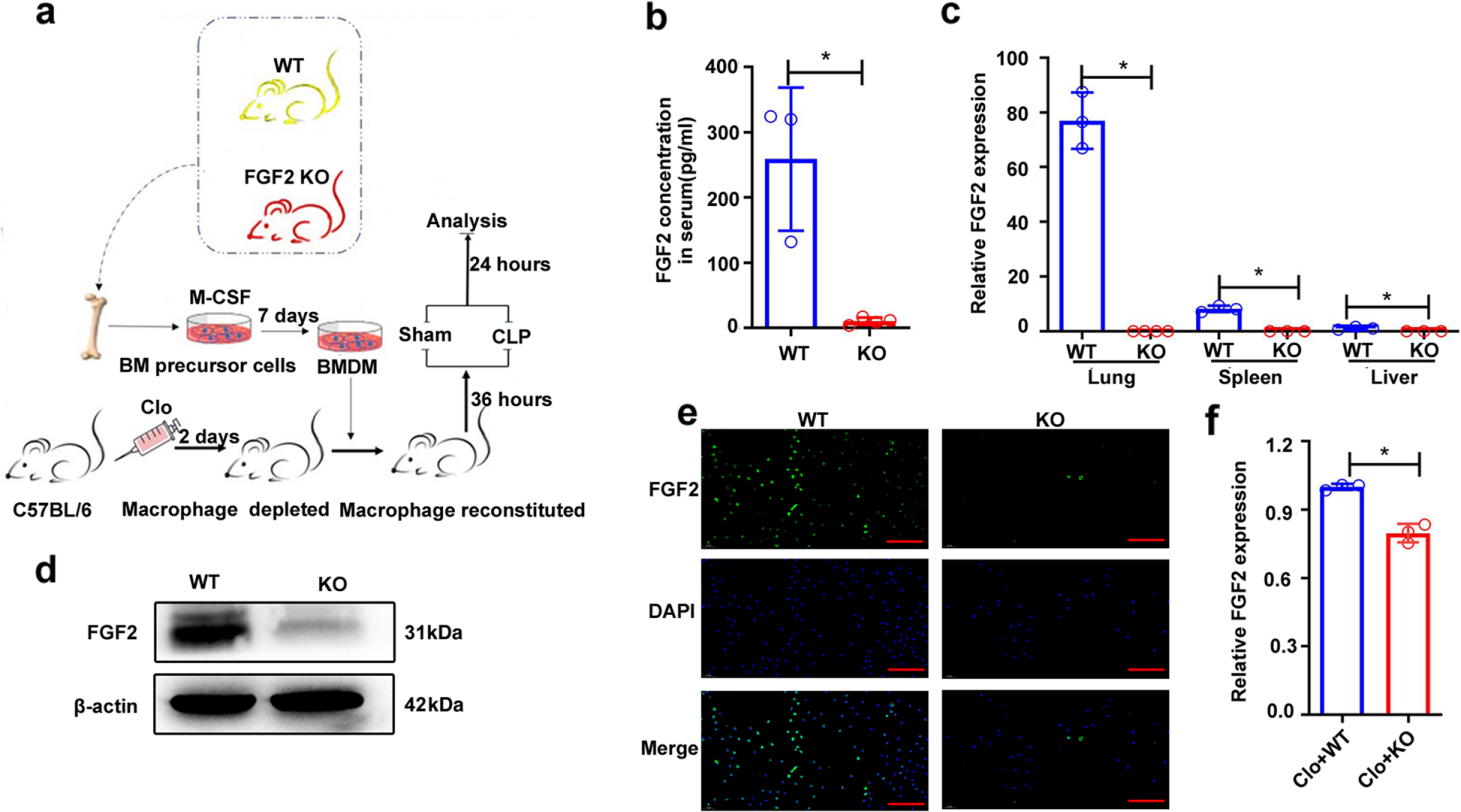
Flow chart of the experimental design and verification of FGF2 deletion. **a** Diagram of experimental design. Bone marrow precursor cells were collected from the tibia and femur of WT and FGF2 KO mice aged 8–12 weeks. They were differentiated into BMDM for 7 days using 100ng/ml M-CSF. Macrophages in C57BL/6 male mice were depleted using clodronate liposomes. BMDM from WT and KO mice were injected via the tail vein into mice to reconstitute macrophages after 2 days. Mice were treated with Sham or CLP, and the samples were analyzed 24 h later. **b** Serum FGF2 protein levels were assessed by ELISA (*n* = 3–5). **c** FGF2 gene expression levels were measured in the lungs, spleen, and liver using real-time PCR (*n* = 3). **d** Western blot analysis of FGF2 protein levels in lung tissue extractions (*n* = 3). **e** Immunofluorescence staining of FGF2 in BMDM from WT and FGF2 KO mice (*n* = 3). **f** The levels of FGF2 gene expression were quantified in the lung tissue of mice subjected to elimination-reconstruction procedures. Bar is 50 μm. *p < 0.05 vs. WT or vs. Clo + WT

The experimental design is illustrated in Fig. 1a. First, we confirmed the effective deletion of FGF2 at both the RNA and protein levels. Serum enzyme linked immunosorbent assay (ELISA) showed that FGF2 was barely present in the serum of FGF2 KO mice, whereas wild-type mice had FGF2 levels exceeding 100pg/ml (Fig. 1b). Real-time PCR showed that FGF2 was most highly expressed in the lungs of WT mice, whereas FGF2 KO mice showed no detectable expression in the lungs, spleen, or liver (Fig. 1c). Western blot analysis of lung tissue revealed a notable reduction in FGF2 expression in FGF2 KO mice (Fig. 1d), whereas immunofluorescence analysis confirmed the lack of FGF2 expression in BMDM isolated from FGF2 KO mice (Fig. 1e). In addition, downregulation of FGF2 expression was also observed in the lungs from elimination-reconstruction mice (Fig. 1f).

**Fig. 2:**
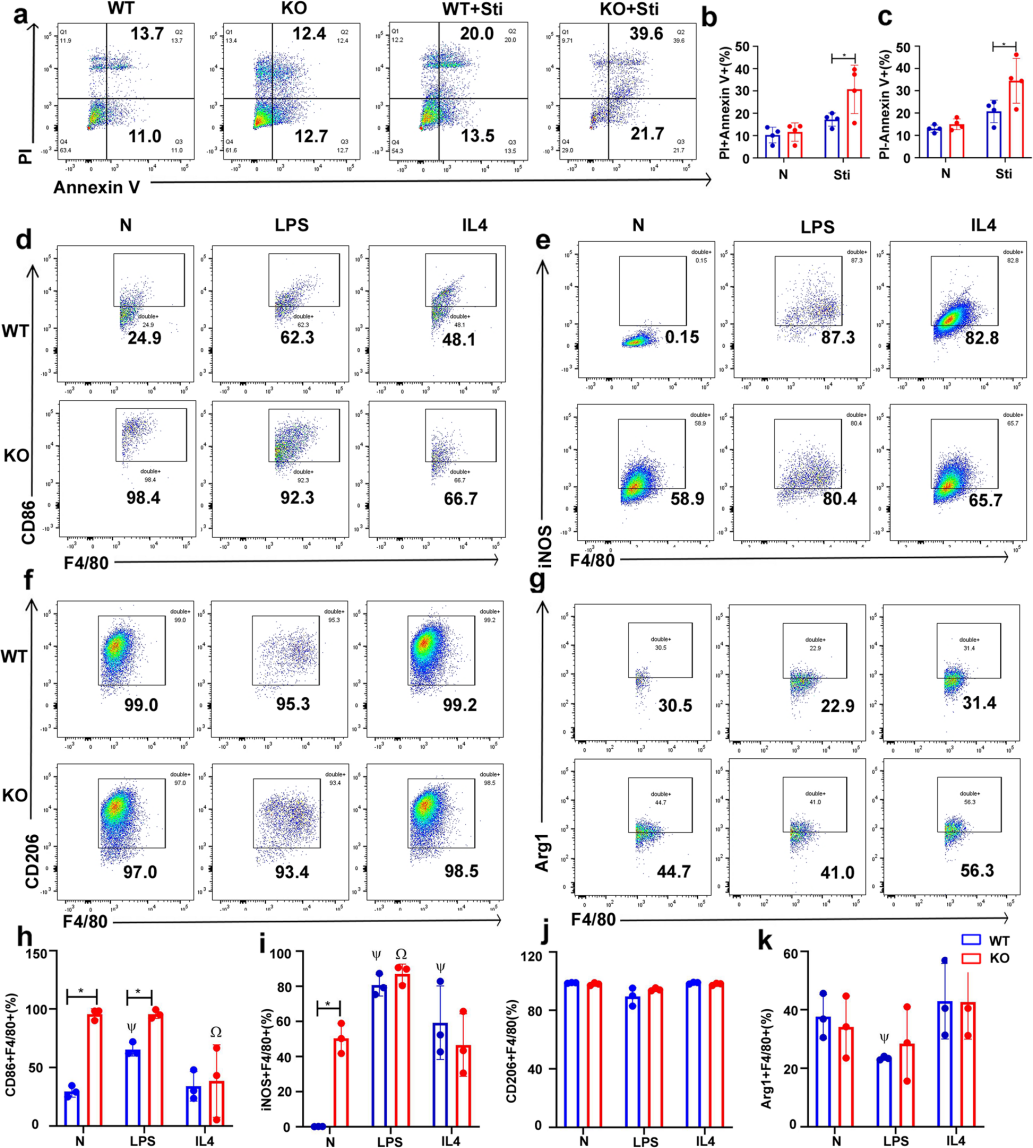
Effect of FGF2 deficiency on BMDM apoptosis and polarization. **a**–**c** FGF2 deletion increased BMDM apoptosis. **a** Apoptosis in BMDM deprived of FBS for 24 h was assessed by flow cytometry (*n* = 4). **b**-**c** Percentage of PI + Annexin V + and PI- Annexin V + BMDM after starvation. **d**-**k** FGF2 deletion in BMDM promoted M1 polarization. **d**-**g** Flow cytometric analysis of macrophage markers in BMDM treated with LPS or IL4, including CD86, iNOS, CD206, and Arg1 (*n* = 3). **h**-**k** The levels of CD86, iNOS, CD206 and Arg1 in BMDM after treatment with LPS or IL4. N represents no treatment; * *p* < 0.05, vs. WT; Ψ *p* < 0.05, vs. N + WT; Ω *p* < 0.05, vs. N + FGF2 KO

To assess the influence of endogenous FGF2 on macrophage function, BMDM were obtained from WT and FGF2 KO mice and the effects of FGF2 deficiency on macrophage apoptosis and polarization were analyzed. Flow cytometry analysis revealed that FGF2 KO macrophages exhibited an increased susceptibility to apoptosis under nutrient deprivation (Fig. 2a-c). CD86 and iNOS are M1 macrophages markers. Flow cytometry analysis showed that CD86 and iNOS increased significantly after FGF2 deletion, and the expression of CD86 in LPS-treated FGF2 KO cells was still higher than that in LPS-treated WT (Fig. 2d, e). CD206 and Arg1, markers of M2 macrophages, were unaffected by FGF2 deficiency (Fig. 2f, g). The findings from the quantitative flow cytometry analysis demonstrated a significant increase in the polarization of BMDM to the M1 type after FGF2 deletion (Fig. 2h-k). Specifically, the percentage of CD86-positive cells increased from approximately 30% to > 95% (Fig. 2h), whereas iNOS expression increased from negligible to > 50% (Fig. 2i). Flow cytometry analysis indicated that the deletion of FGF2 enhanced the differentiation of BMDM toward the M1 phenotype. In addition, when FGF2 KO BMDM were treated with IMD0354, which suppresses NF-κB, or MCC950, a selective inhibitor of NLRP3 inflammasomes, there was a noticeable decrease in the percentage of iNOS (Fig. S1).

### FGF2 deficiency in macrophage resulted in elevated expression of proinflammatory cytokines and an enhanced inflammatory response

**Fig. 3:**
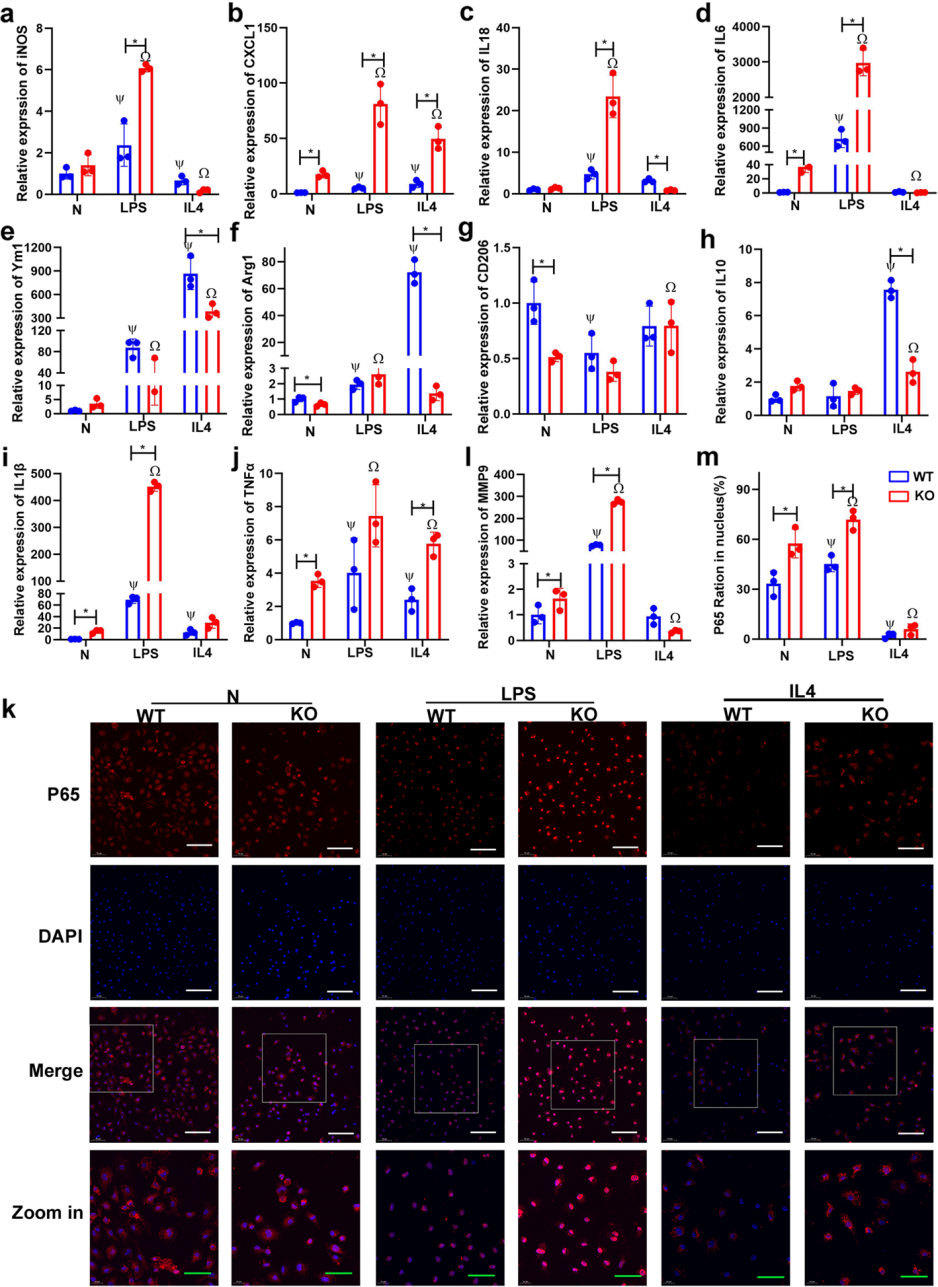
Deficiency of FGF2 in BMDM resulted in the upregulation of M1 markers and proinflammatory cytokine expression and increased nuclear translocation of NF-KB p65. **a**-**j** BMDM were treated with LPS or IL4, and real-time PCR was used to determine the expression of M1 and M2 markers and cytokines (*n* = 3). **k**, **l** P65 nuclear translocation was detected and quantitatively analyzed by immunofluorescence (*n* = 3). **m** The expression of MMP9 in WT and FGF2 KO BMDM treated with LPS or IL4 were determined with real-time PCR. Bar in the first three rows is 50 μm, while bar in the fourth rows is 20 μm, * *p* < 0.05 vs. WT; Ψ *p* < 0.05 vs. N + WT; Ω *p* < 0.05 vs. N + KO

Cytokine expression was evaluated in BMDM from FGF2 KO and WT mice following exposure to polarization stimuli: LPS for M1 and IL4 for M2. After LPS stimulation, the expression levels of iNOS, CXCL1, IL18, IL6, IL1β, and TNFα were significantly upregulated in both WT and FGF2 KO, with FGF2 deficiency demonstrating significantly higher expression levels than WT (Fig. 3a-d, i, j). Without stimulation, BMDM derived from FGF2 KO mice exhibited higher expression levels of CXCL1, IL6, IL1β, and TNFα (Fig. 3b, d, i, j). Additionally, BMDM derived from FGF2KO mice showed a significant decrease in CD206 levels, whereas both WT and FGF2KO BMDM showed a substantial reduction in CD206 levels upon exposure to LPS (Fig. 3g). Surprisingly, an increase in Ym1 (M2 marker) expression was observed following LPS stimulation, although its level was notably lower than that in IL4 stimulation (Fig. 3e). Upon IL4 treatment, the M2 macrophage-associated genes Ym1, Arg1, and IL10 were significantly upregulated, and their expression levels in FGF2 KO macrophages were notably lower than those in WT macrophages (Fig. 3e, f, h). Additionally, IL4-treated BMDM exhibited a shift towards M2 polarization, while the levels of the inflammatory cytokines CXCL1 and TNFα were still significantly higher than those in WT BMDM (Fig. 3b, j). These findings indicate that the deficiency of FGF2 may contribute to the promotion of M1 polarization and upregulation of proinflammatory cytokine expression.

To further explore the effect of FGF2 deletion on the inflammatory response, immunofluorescence was used to assess nuclear translocation of p65 NF-κB in BMDM (Fig. 3k). In the absence of stimulation, the percentage of nuclear localization of p65 in the FGF2 KO group was notably greater than that in the WT group (Fig. 3k, l). Following LPS stimulation, both WT and FGF2 KO macrophages exhibited a significant increase in fluorescence intensity and p65 proportion, however, the expression intensity of p65 NF-κB in FGF2 KO macrophages was stronger than that in WT macrophages (Fig. 3k, l). Following polarization to M2 induced by IL4 treatment, the expression of p65 and nuclear translocation of WT and FGF2 KO BMDM decreased significantly (Fig. 3k, l). Matrix metalloproteinase (MMP) 9 regulates macrophages polarization to M1 by activating p65 [16]. Therefore, we examined the effect of FGF2 deletion on MMP9 expression in macrophages. In the absence of stimulation, the expression of MMP9 increased after FGF2 knockout in BMDM (Fig. 3m). LPS stimulation promoted the expression of MMP9, and the expression level in FGF2 KO cells was significantly higher than that in WT cells (Fig. 3m). Our results show that FGF2 knockout in BMDM affects the expression of inflammatory cytokines and the nuclear translocation of p65 NF-κB, as well as the cellular response to LPS and IL4 stimulation.

### Transcriptome sequencing analyses of BMDM from FGF2 KO and WT mouse upon LPS stimulation

**Fig. 4:**
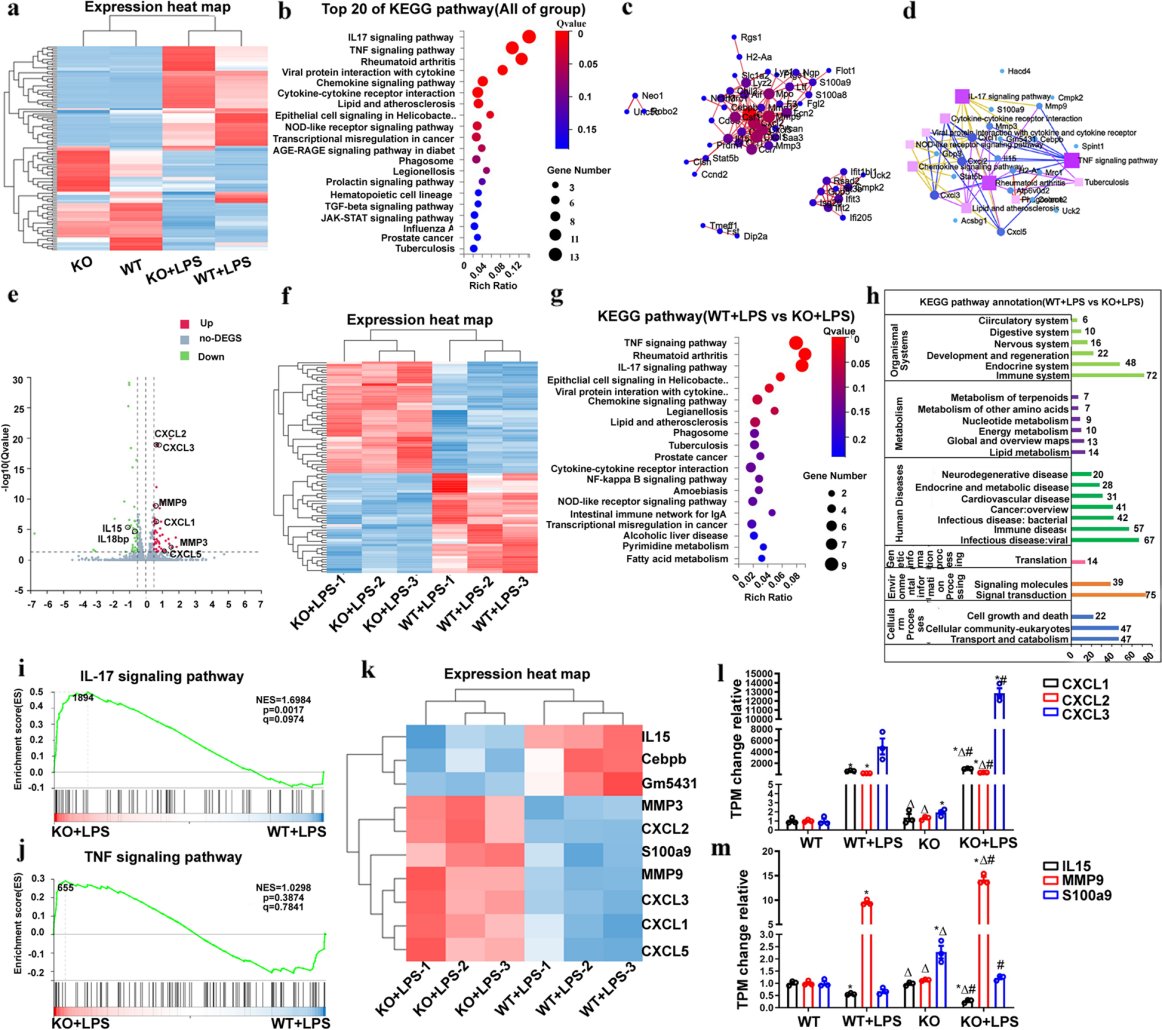
Transcriptome sequencing was used to analyze BMDM from WT and FGF2 KO mice treated with or without LPS. **a** Heat map of DEGs in BMDM from WT and FGF2 KO mice stimulated with or without LPS (Average TPM each group, *n* = 3). **b** KEGG enrichment analysis identified the top 20 altered signaling pathways in the four groups (WT, FGF2 KO, WT + LPS, and FGF2 KO + LPS). **c** Construction of a regulatory network to modulate PPI involving FGF2 and LPS using DEGs. **d** KEGG pathway network based on similarity in gene expression profiles. **e** Volcano plot showing DEGs of WT + LPS and FGF2 KO + LPS. **f** Heat map of DEGs in BMDM of WT + LPS and FGF2 KO + LPS. **g** Top 20 KEGG pathways for DEGs in BMDM from WT + LPS and FGF2 KO + LPS. **h** KEGG pathway annotation of differentially expressed genes between WT + LPS and FGF2 KO + LPS. **i**, **j** Differently regulated pathways in the GSEA. **k** Inflammation and cytokine gene heat map for WT + LPS and FGF2 KO + LPS. **l**, **m** TPM changes in the gene groups compared to WT weights. * *p* < 0.05 vs. WT; Δ *p* < 0.05 vs. WT + LPS; # *p* < 0.05 vs. FGF2 KO

To elucidate the biological mechanism underlying the effect of FGF2 deletion on macrophages, RNA sequencing was performed on BMDM from WT and FGF2 KO mice with and without LPS treatment. A total of 125 genes exhibiting differential expression (log2 fold change > 0.5, Q value < 0.05) were identified and used to construct a gene expression heat map (Fig. 4a). Kyoto Encyclopedia of Genes and Genomes (KEGG) enrichment analysis was performed to determine the top 20 signaling pathways that demonstrated a significant influence (Fig. 4b). A PPI regulatory network map was constructed using the differentially expressed genes, and the KEGG pathway network map was based on the similarity of the gene expression profile (Fig. 4c, d). Subsequently, following LPS treatment, 89 genes in WT and FGF2 KO mice showing differential expression (log2 fold change > 0.5, Q value < 0.05) were identified and subjected to analysis to generate a volcano plot, differential gene heat map, top 20 signaling pathways enriched by KEGG analysis, and KEGG annotation map (Fig. 4e-h). Figure 4i and j depict the signal pathway diagram of IL17 and TNFα obtained through GSEA, whereas Fig. 4i and m show the TPM expression histogram of genes involved in these signaling pathways.

### Macrophage FGF2 deficiency aggravates CLP-induced septic acute lung injury

**Fig. 5:**
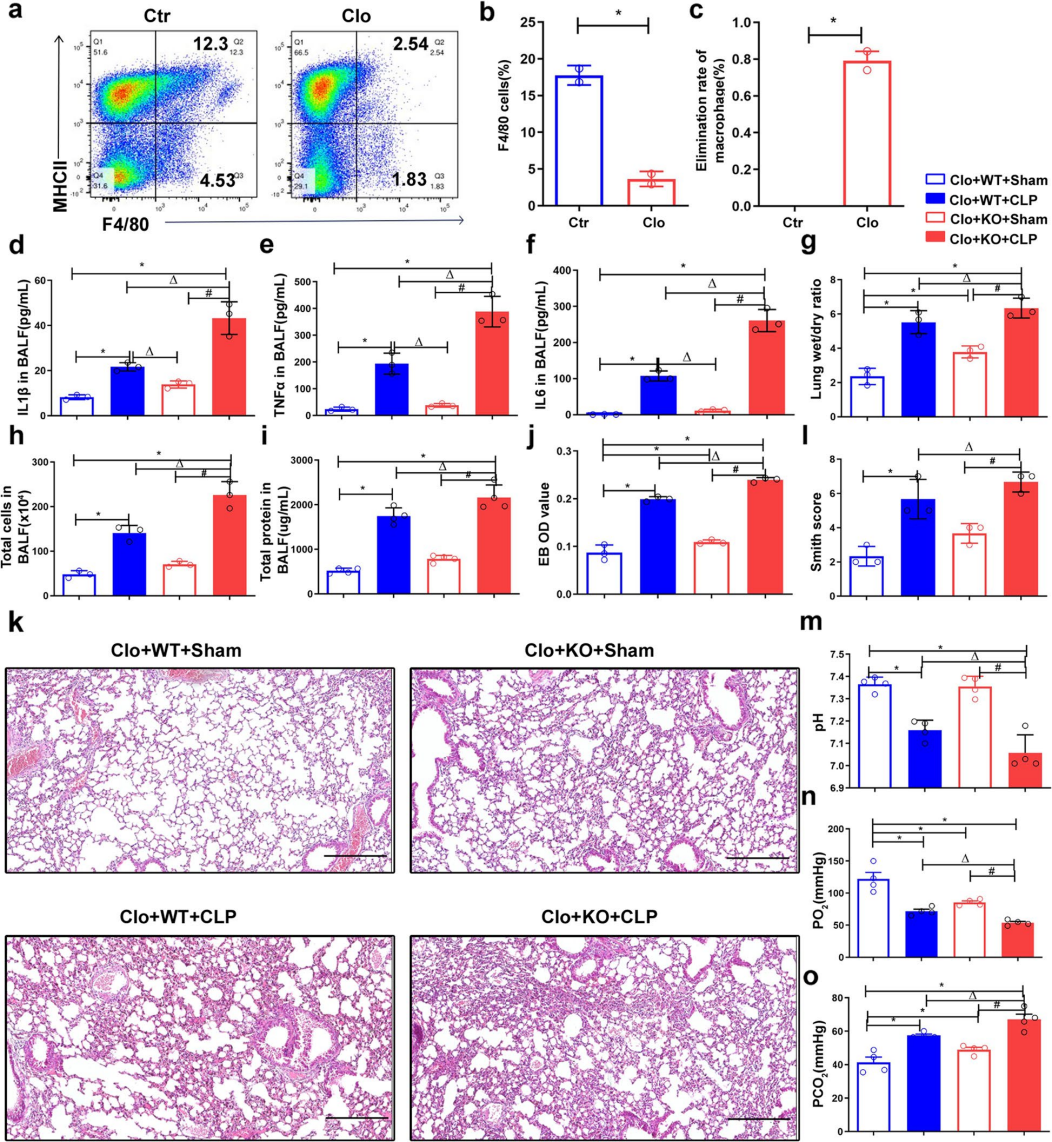
FGF2 deletion in macrophages aggravates lung injury in CLP mice. **a**-**c** Clodronate liposomes were injected intravenously to deplete macrophages, followed by quantification of F4/80 + spleen cells using flow cytometry to measure macrophage clearance. *p < 0.05 vs. control. **d**-**f** Levels of the inflammatory cytokines IL1β, TNFα, and IL6 in bronchoalveolar lavage fluid (BALF) from the four groups were quantified via ELISA. The four groups were Clo + WT + Sham, Clo + WT + CLP, Clo + FGF2 KO + Sham, and Clo + FGF2 KO + CLP. **g** The lung wet-to-dry weight ratios were measured in the four groups. **h**-**i** The total cells and total protein in BALF were evaluated in the four groups. **j**The OD value of Evans blue in the lung tissues from the four groups. **k**-**l** HE staining of lung tissue from the four groups and their Smith score. (m-o) Blood gas analysis was performed using abdominal aortic blood from the four groups. Bar is 250 μm. * *p* < 0.05 vs. WT; Δ *p* < 0.05 vs. WT + LPS; # *p* < 0.05 vs. FGF2 KO

To investigate the in vivo role of FGF2 deficiency macrophages, we used a macrophage clearance and reconstruction model, in conjunction with CLP surgery, to induce sepsis and assess the impact of FGF2 deletion on septic ALI. Referring to methods described in the literature [17], we first evaluated the clearance efficiency of endogenous macrophages. Mice were administered clodronate liposomes, followed by the extraction of spleen cells for analysis of the F4/80 + cell proportion using flow cytometry (Fig. 5a, b). The results indicated that singular administration resulted in approximately 80% depletion of macrophages (Fig. 5c). BMDM from WT or FGF2 KO were intravenously injected to reconstitute the immune system. In vivo imaging experiments were performed to illustrate the successful transplantation of cells into mice through tail vein injection (Fig. S2). The concentrations of inflammatory cytokines IL1β, TNFα, and IL6 in bronchoalveolar lavage fluid (BALF) were analyzed using ELISA 24 h after CLP (Fig. 5d-f). The results demonstrated a significant increase in the expression of these inflammatory factors in the CLP group compared with that in the sham group. Additionally, the expression of IL1β, TNFα, and IL6 in BALF from FGF2 KO mice was higher than that in WT mice in the sepsis model (Fig. 5d-f). Consistently, the total cell number, protein concentration, and lung wet-to-dry weight ratio in BALF were significantly higher after CLP surgery (Fig. 5g-i). In addition, the average value in the FGF2 KO group was significantly higher than that in the WT group in the sepsis model (Fig. 5g-i). Evans blue OD value, indicative of lung tissue permeability, was significantly higher in the FGF2 KO group than in the WT group following CLP surgery (Fig. 5j). The lung wet-to-dry weight ratio and Evans blue OD value of FGF2 KO mice in the sham group were significantly higher than those in the WT sham group (Fig. 5g, j). Histological examination of lung tissue following CLP-induced sepsis revealed deterioration of lung injury (Fig. 5k). Additionally, the Smith score (lung injury score) demonstrated a significant increase in the CLP group compared with the sham group, whereas the FGF2 KO group exhibited a significant increase compared to the WT group following CLP (Fig. 5l). Furthermore, the results of the blood gas analysis indicated that the pH and pO_2_ levels were significantly lower in the CLP group than in the sham group, whereas the pCO_2_ levels were significantly higher in the CLP group than in the sham group (Fig. 5m-o). In addition, there were significant differences in pH, pO_2_, and pCO_2_ levels between WT and FGF2 KO mice after CLP (Fig. 5m-o). These results suggest that the deletion of FGF2 in macrophages aggravates acute lung injury in sepsis.

### Increased M1 macrophage polarization and apoptosis in the lungs of CLP mice following reconstitution with FGF2 KO macrophages

**Fig. 6:**
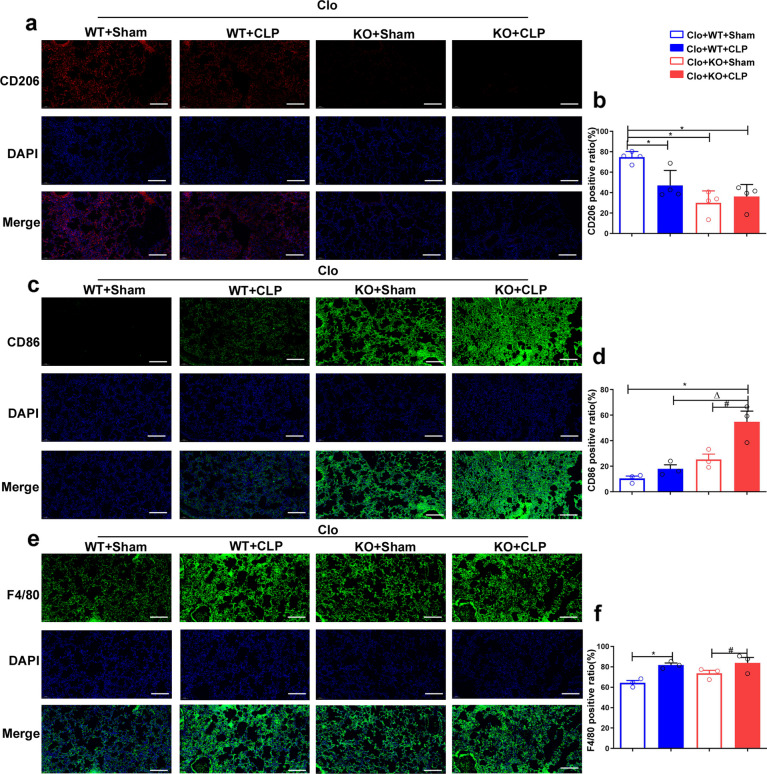
Mice reconstituted with FGF2 KO macrophages and subjected to CLP demonstrate increased M1 polarization in lung tissue. **a**-**f** The presence and levels of CD206, CD86, and F4/80 markers on macrophages within lung tissue were identified and quantitatively assessed using immunofluorescence staining. Bar is 20 μm. * *p* < 0.05, vs. WT; Δ *p* < 0.05 vs. WT + LPS; # *p* < 0.05 vs. FGF2 KO

To elucidate the intricate cellular and molecular mechanisms by which FGF2 knockout in macrophages aggravates septic acute lung injury, immunofluorescence was used to assess the expression levels of the M1 macrophage marker CD86, M2 macrophage marker CD206, and macrophage marker F4/80 (Fig. 6a, c, e). Quantitative analysis was conducted to evaluate these markers. In the sham group, there was a significant decrease in the CD206 positive rate after FGF2 KO transplantation (Fig. 6b). The CD86 positive rate in lung tissue following CLP was significantly increased, with a more pronounced increase observed in FGF2 KO mice than in WT mice (Fig. 6c, d). Following CLP surgery, there was a significant increase in the number of F4/80 positive macrophages in both WT and FGF2 KO groups (Fig. 6e, f).

**Fig. 7:**
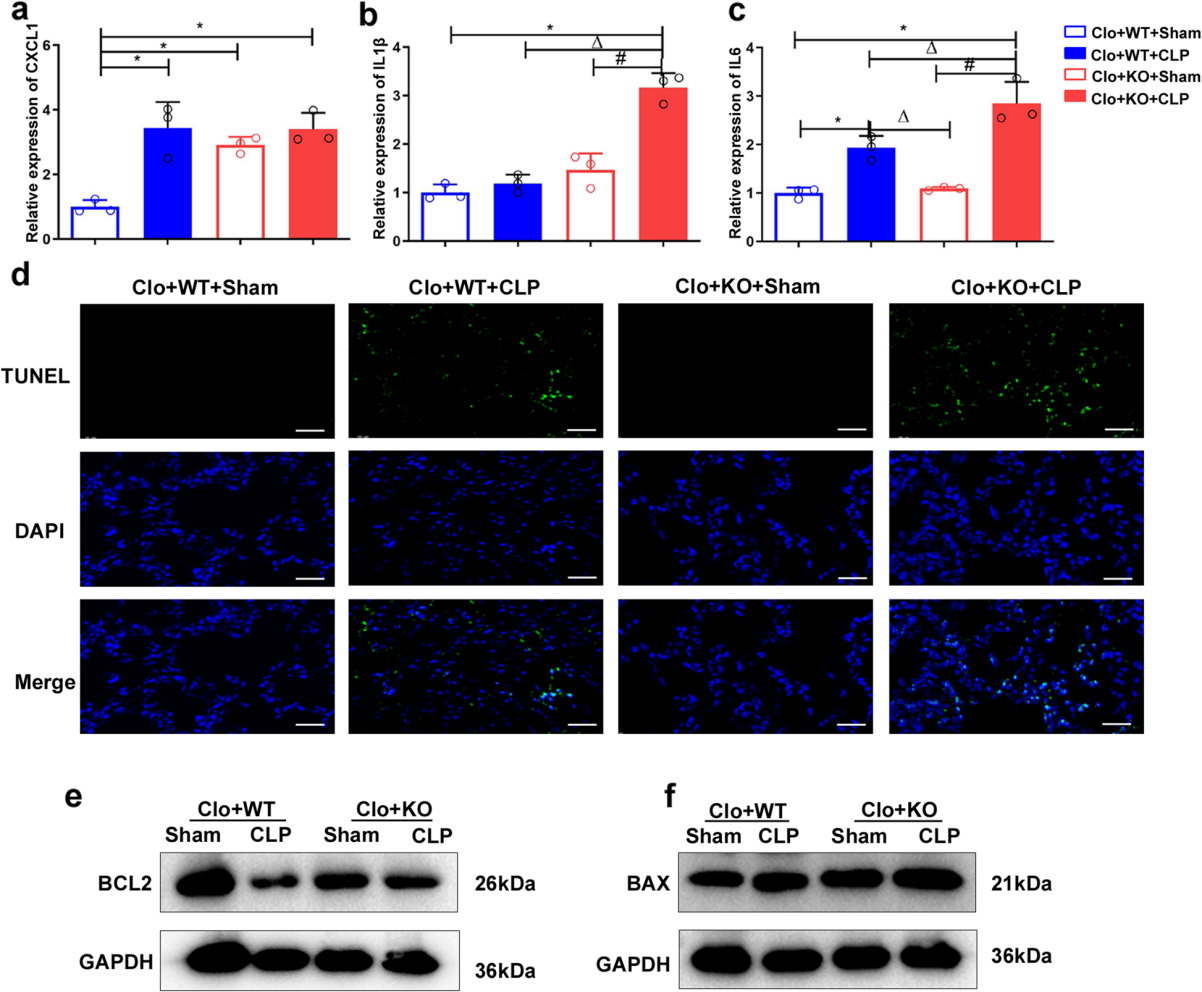
Mice reconstituted with FGF2 KO macrophages and subjected to CLP exhibit increased proinflammatory gene expression and apoptosis. **a**-**c** The expression of the inflammatory factors CXCL1, IL1β, and IL6 in lung tissue was detected by real-time PCR (*n* = 3 per group). **d** TUNEL staining was used to evaluate apoptosis in the lung tissue. **e**-**f** The apoptosis-associated genes BCL2 and Bax were identified by western blot analysis. Bar is 20 μm. * *p* < 0.05, vs. WT; Δ *p* < 0.05, vs. WT + LPS; # *p* < 0.05 vs. FGF2 KO

Real-time PCR was used to assess the expression of the inflammatory factors CXCL1, IL1β, and IL6 in lung tissue (Fig. 7a-c). In the sham group, the expression of CXCL1 in the lung tissue of FGF2 KO mice was higher than that in WT mice (Fig. 7a). However, following CLP surgery, a significant increase in CXCL1 was observed in the WT group, while a significant increase in IL1β was noted in the FGF2 KO CLP group compared to that in the FGF2 KO sham group (Fig. 7a, b). The study revealed a significant increase in the expression level of IL6 in the CLP group compared to the sham group, as well as in the FGF2 KO group compared to the WT group after CLP surgery (Fig. 7c). This study indicates that the absence of FGF2 in macrophages promotes M1 polarization and increases the expression of inflammatory mediators in the lung tissue, potentially exacerbating pathological damage in the lungs.

Subsequently, the TUNEL assay was employed to identify apoptotic cells in the lung tissue of each experimental group. Our analysis revealed a notable increase in the number of TUNEL -positive cells in CLP-induced septic lung tissue compared to that observed in the sham group of the same genotype (Fig. 7d). Additionally, following CLP surgery, the number of TUNEL positive cells observed in the FGF2 KO transplant group exceeded that in the WT group (Fig. 7d). Furthermore, aberrant expression of inflammasome-related markers, specifically NLRP3, ASC, and Caspase-1 p20, was observed in the lungs of the CLP group transplanted with FGF2 KO macrophages (Fig. S3). Western blot analysis of lung tissue proteins revealed a notable decrease in the anti-apoptotic protein BCL2 after CLP surgery in mice of the same genotype (Fig. 7e). In the sham group, a decrease in BCL2 expression was noted in the FGF2 KO transplant group compared to the WT transplant group, whereas an increase in BAX expression was observed in the FGF2 KO transplant group compared to the WT transplant group (Fig. 7e, f). We hypothesized that increased apoptosis induced by FGF2 deficiency in macrophages contributes to the exacerbation of pathological damage.

## Discussion

This study examined the impact of FGF2 deletion on macrophage function in vitro and septic ALI in vivo. This is the first study to demonstrate that deletion of FGF2 in macrophages promotes macrophage polarization towards the M1 phenotype, enhances the expression of inflammatory mediators, and exacerbates lung pathology in CLP-induced sepsis.

Extensive evidence suggests that studying macrophage polarization has great potential for the treatment of sepsis-induced lung injury [9]. However, the mechanisms governing macrophage polarization during the progression of septic acute lung injury (ALI) are not fully understood. Previous research has demonstrated a significant association between the FGF signaling pathway and macrophage polarization [18]. Specifically, FGFR2 has been identified as a driver of M2 macrophage polarization in colorectal cancer by increasing PAI-1 expression via the JAK2/STAT3 pathway [19]. Additionally, FGF20 has been found to reduce inflammation in macrophages through its interaction with FGF receptor 1, leading to a decrease in M1 macrophage markers and proinflammatory cytokines [20]. Notably, the same FGF may exert differential effects on macrophage polarization depending on the context. In nasopharyngeal cancer survivors, elevated FGF2 levels in tissues activate CXCL14, promoting M2 macrophage polarization and tumor metastasis [21]. Conversely, in patients with rheumatoid arthritis, increased FGF2 expression induces M1 macrophage polarization and inflammatory factor release, causing joint damage [22]. In this study, a significant increase in the percentage of iNOS and CD86 markers in BMDM from FGF2 KO mice was detected using flow cytometry. There was also a significant increase in the rate of CD86 immunofluorescence positivity in the lung tissues of CLP-septic mice after reconstitution with FGF2 knockout macrophages. Our results indicated that a lack of FGF2 in macrophages facilitated polarization towards the M1 phenotype.

In accordance with the present findings, it has been demonstrated that deletion of the low-molecular-weight FGF2 isoform can lead to the polarization of macrophages toward the M1 phenotype [13]. FGF2 is composed of various protein isoforms, including low-molecular-weight (LMW) and high-molecular-weight (HMW) variants, which are generated through alternative translation of the FGF2 gene. The distinct localization of these isoforms within various cellular compartments suggests that they may exhibit unique biological activity. Notably, differential effects between the LMW and HMW isoforms of FGF2 have been documented in the context of liver fibrosis and osteoarthritis development [23, 24]. However, it has been reported that both the HMW 34-kDa isoforms and the LMW 18-kDa mature FGF2 isoforms exhibit identical roles in the induction of VEGF [25]. To the best of our knowledge, the effect of HMW isoforms of FGF2 on macrophages has not been reported and whether HMW isoforms have identical effects on M1 macrophage polarization remains to be further explored.

Macrophage polarization is governed by a complex regulatory network that is modulated by an array of signaling molecules, transcription factors, epigenetic modifications, and metabolic reprogramming [26]. Recent studies have demonstrated that certain transcription factors are critical to macrophage polarization [27, 28]. In our study, we observed a significant increase in both the proportion of NF-κB translocated into the nucleus of bone marrow-derived macrophages (BMDMs) and the mRNA expression levels of MMP9 in FGF2-deficient macrophages. Flow cytometric analysis revealed a significant reduction in iNOS -positive M1 macrophages in FGF2 knockout BMDMs subjected with an inhibitor of NF-κB pathway. Previous research has demonstrated the significance of NF-κB in facilitating the M1 polarization of RAW 264.7 macrophages in the tumor microenvironment, as well as in guiding the polarization of macrophages towards the M1 phenotype in Behçet’s disease patients [29, 30]. Consistent with the findings of our study, previous research has indicated the pivotal role of MMP9 in macrophage phenotypic switching. Specifically, recombinant MMP9 has been shown to exacerbate LPS-induced M1 switching by activating NF-κB, while small interfering RNA targeting MMP9 inhibits the LPS-mediated M1 phenotype [16]. Furthermore, it has been reported that the deletion of MMP9 facilitates M2 macrophage polarization, whereas the migration of inflammatory macrophages requires the activation of MMP9 in murine models [31, 32]. Therefore, we infer that the lack of FGF2 possibly switches the M1 polarization of macrophages by modulating NF-κB signaling mediated by MMP9.

Previous research has identified M1 macrophages as crucial in the inflammatory response due to their secretion of pro-inflammatory cytokines and chemokines [33]. Moreover, researchers have discovered a connection between FGF2 and inflammatory pathways [34]. The results of our study suggest that the lack of FGF2 in macrophages results in the increased expression of inflammatory cytokines, specifically IL1β and TNFα, in vitro. RNA-seq analysis indicated that the differential expression of genes in macrophages following FGF2 deletion was primarily enriched in two inflammatory pathways, specifically the IL17 and TNFα signaling pathways. Following well-established methods [17], mice depleted of endogenous macrophages reconstructed with FGF2 KO macrophages, combined with CLP surgery, showed a significant increase in the mRNA expression of IL1β and IL6 in the lung tissue, along with elevated protein levels of IL1β and TNFα in the bronchoalveolar lavage fluid of the FGF2 KO group. The results of this study demonstrated the important role of FGF2 in regulating inflammatory responses by macrophages. These findings are consistent with those observed in earlier studies, which showed a strong association between FGF signaling and inflammation and lung injury. FGF10 has been shown to mitigate lung-specific inflammation resulting from traumatic or infectious lung injury [35]. Furthermore, pretreatment with FGF1 or rFGF4 has been demonstrated to confer protection against LPS-induced ALI by modulating anti-inflammatory and antioxidant pathways [36, 37]. FGF21 suppresses inflammatory responses in LPS-induced ALI by regulating the TLR4/MYD88/NF-κB signaling pathway [38]. Our analysis revealed that the loss of FGF2 in macrophages triggered M1 polarization and heightened the expression of inflammatory cytokines, which may potentially contribute to the exacerbation of CLP-induced lung tissue damage in the FGF2 KO macrophage transplantation group.

Recently, substantial progress has been made in elucidating the role of the NLRP inflammasome in both health and disease. Moreover, dysregulated activation of the NLRP inflammasome has been critically implicated in the pathogenesis of acute lung injury and pneumonia [39, 40]. In our study, increased IL1β in BALF was observed in mice transplanted with FGF2 KO macrophages, and flow cytometry showed that NLRP3 inhibitor treatment significantly decreased the proportion of M1 macrophages in FGF2 knockout BMDM. Aberrant expression of NLRP3, ASC and Caspase-1 p20 was observed in the lungs of mice in the CLP group transplanted with FGF2 KO macrophages. Based on these preliminary results, we inferred that the NLRP3 inflammasome may play a partial role in the polarization of M1 macrophages in FGF2 knockout macrophages and sepsis-induced acute lung injury. Next, we will explore the mechanism by which FGF2 controls the activation of the NLRP3 inflammasome in greater detail.

In conclusion, this study used BMDM obtained from FGF2 KO mice to demonstrate that FGF2 deletion enhances the M1 polarization of macrophages and upregulates the production of inflammatory mediators. Subsequent macrophage depletion and reconstitution experiments in sepsis mouse models have provided evidence that FGF2 KO macrophages aggravated sepsis-induced acute lung injury. To the best of our knowledge, this is the first instance in which FGF2 has been shown to control macrophage M1 polarization, and the removal of FGF2 from macrophages exacerbates septic acute lung injury. Our study offers empirical evidence that can aid in understanding the development of septic lung injury and the potential benefits of modulating FGF2 signaling to regulate macrophage polarization as a means to mitigate this condition.

## Materials and methods

### Mice and ethics statement

FGF2 KO mice obtained from GemPharmatech Biotechnology Co. Ltd. (China) were housed and bred at the Animal Experiment Center of the Fourth Medical Center of the PLA General Hospital. The FGF2 gene in these mice was modified using CRISPR/Cas9 technology, specifically targeting exon 2 of the FGF2-208 (ENSMUST00000200585.4) transcript. The region contains a 104 bp coding sequence, and deletion of this region results in loss of protein function. All mice used in the study were of the C57BL/6 background and were aged between 8 and 12 weeks. All animal experiments conducted in this study were approved by the Animal Experiment Center of the People’s Liberation Army General Hospital (approval ID: IACUC-2023-0010), and all procedures were performed in compliance with established international guidelines for animal research.

### Method for BMDMs isolation and culture

BMDM were isolated according to previously established methods [17]. Briefly, tibia and femur were obtained from WT and KO mice aged 8–12 weeks. The bone marrow cells were washed with PBS (Cat No. G4202-500mL, Servicebio, Wuhan, China), and erythrocyte lysis was performed using red blood cell lysis solution (Cat No. R1010, Solarbio Science and Technology, Beijing, China). The cells were cultured in RPMI 1640 medium (Cat No.10-040-CV, CORNING, NY, USA) supplemented with 10% FBS (Cat No.A5669701, Gibco, ThermoFisher Scientific, CN), 1% penicillin-streptomycin (Cat No.P4333, BasalMedia, Shanghai, China), and 100ng/ml M-CSF (Cat No. Z03275, GenScript, Nanjing, China) at 37 °C in a humidified 5% CO2 sterile incubator for seven days. Subsequently, BMDM were cultured in fresh medium containing 100ng/ml LPS (Cat No.L4391, E. coli 0111:B4, Sigma-Aldrich, USA) for M1 polarization or in fresh medium containing 10ng/ml IL4 (Cat No.SRP3211, Sigma-Aldrich, USA) for M2 polarization, in preparation for further experiments.

### Flow cytometry

BMDM were exposed to LPS at a concentration of 10 ng/ml for 48 h to induce M1 polarization, and IL4 at a concentration of 10 ng/ml for 48 h to induce M2 polarization. Subsequently, the cells were digested with trypsin (Cat No. BL512B, Labgic, Beijing, China), resuspended in precooled PBS, and analyzed for BMDM polarization by flow cytometry. The primary antibody used to identify M1 macrophages was FITC-conjugated anti-mouse CD86 (0.125 µg/test, Cat No. Cat No.11-0862-81, Invitrogen, USA) and PE-conjugated anti-mouse inducible nitric oxide synthase (iNOS) (0.06 µg/test, Cat No. 12-5920-82, Invitrogen, USA). To identify M2 macrophages, APC-conjugated anti-mouse Arg-1 (1 µg/test, Cat No. 17-3697-80, Invitrogen, USA) and PE-conjugated anti-mouse CD206 (0.125 µg/test, Cat No. 12-2061-80, Invitrogen, USA) were used. FITC-conjugated anti-mouse F4/80 (0.05 µg/test, Cat No. 123108, Biolegend, USA) was used for macrophage identification. Following antibody labeling, the samples were subjected to flow cytometry analysis using BD Cantoll, and the data were processed using the Diva software. In addition, BMDM from KO and WT mice were treated with 1.2 µM IMD-0354 (IKK2 inhibitor V, Cat No.HY-10172, MCE, USA) for 3 h or with 10 nM MCC950 (a selective inhibitor of NLRP3, Cat No. HY-12815, MCE, USA) for 10 h, and iNOS expression was detected by flow cytometry.

For apoptosis assessment, BMDM were subjected to nutrient deprivation in a medium containing 1% FBS for 24 h. Subsequently, BMDM apoptosis was evaluated using Annexin V/PI apoptosis detection kits (Cat No. KGA1101-20, KeyGEN Biotech, Nanjing, China) using flow cytometry following the manufacturer’s protocol. Briefly, cells were digested with trypsin and counted using a hemocytometer. A total of 5 × 10^5^ cells were collected per tube and resuspended in 500 µL binding buffer. Single-cell suspensions were incubated with 5 µL Annexin V-FITC and 5 µL PI for 5–10 min. The apoptosis rate of cells was measured using a flow cytometer.

### Immunofluorescence staining of BMDM and lung tissue

Immunofluorescence staining of BMDM was performed as described previously [10]. Briefly, BMDM were stimulated with 10 ng/ml LPS or 10 ng/ml IL4 for 24 h. After fixation with 4% paraformaldehyde (Cat No.BL539A, Labgic, Beijing, China) for 10 min, the cells were blocked with normal goat serum (Cat No. A7007, Beyotime, Jiangsu, China) for 30 min at room temperature, followed by incubation with either FGF2 antibody (1:100, Cat No. MA00121, Boster, Wuhan, China) or P65 antibody (1:500, Cat No.8242, CST, USA) for 1 h at 37 °C. Subsequently, the cells were incubated with fluorescently labeled secondary antibodies (goat anti-rabbit IgG, 1:200, Cat No.BA1054, Boster, Wuhan, China) at 37 °C for 1 h. DAPI (Cat No.HY-D0814, MedChemExpress, NJ, USA) staining was performed for 5 min. Lung tissues were embedded in paraffin, sectioned into 5 μm thick slices, deparaffinized, and rehydrated for immunofluorescence staining. The tissue sections were incubated overnight at 4℃ with primary antibodies, including the rabbit polyclonal antibody CD206 (1:200, Cat No. A02285-2, Boster, Wuhan, China), rabbit monoclonal antibody CD86 (1:100, Cat No. BM4121, Boster, Wuhan, China), rabbit polyclonal antibody F4/80 (1:100, Cat No. 29414-1-AP, Proteintech, Wuhan, China), anti-mouse NLRP3 (1:200, Cat No.68102-1-Ig, Proteintech, Wuhan, China), Caspase-1 p20 Rabbit pAb (1:400, Cat No.bs-10743R, Bioss, Beijing, China) and ASC/TMS1 Rabbit PolyAb (1:200, Cat No.10500-1-AP, Proteintech, Wuhan, China). After washing with PBS, the sections were incubated with a secondary antibody (goat anti-rabbit IgG, 1:200, Cat No.BA1054, Boster, Wuhan, China) for 1 h at room temperature. The nuclei were stained with DAPI for 5 min. Cells and lung tissue slice images were captured using a 3D panoramic scanner (Pannoramic Confocal, 3DHISTECH), and P65 nuclear translocation was quantified using the ImageJ software.

### Real-time PCR analysis

RNA from the lung tissue or BMDM was extracted using TRIzol (Cat No. A211-01, Toroivd, Shanghai, China), according to the manufacturer’s instructions. Complementary DNA (cDNA) was synthesized using a reverse transcription kit (Cat No. RTM-002, Toroivd, Shanghai, China) and quantitative polymerase chain reaction (qPCR) was performed using TOROGreen qPCR Master Mix (Cat No. QST-103, Toroivd, Shanghai, China) using a QuantStudio5 detector. The experiments were conducted in triplicate, and the gene expression levels were normalized using the internal reference cyclophilin. The relative expression of the target genes was analyzed using the Quant Studio Design and Analysis desktop software. The primer sequences used for real-time PCR are listed in Supplementary Table 1.

### RNA-seq analysis

BMDM were lysed using TRIzol and subsequently sent to BGI Company (China) for RNA sequencing analysis. BMDM from WT and FGF2 KO mice were treated with LPS for 24 h with or without treatment. Each group consisted of three individual samples. RNA extraction and sequencing were performed at BGI, followed by data analysis, mining, and mapping using BGI’s Dr. Tom multigroup data mining system (https://biosys.bgi.com/). Gene expression levels were determined using RSEM (v1.3.1), and a heatmap was generated using heatmap (v1.0.8) to visualize gene expression variations across different samples. Differential expression analysis was conducted using DESeq2 (v1.4.5) with a Q-value ≤ 0.05. Enrichment analyses of annotated differentially expressed genes were conducted using Gene Ontology (GO) (http://www.geneontology.org/) and Kyoto Encyclopedia of Genes and Genomes (KEGG) (https://www.kegg.jp/) pathways using the Phyper method employing a hypergeometric test. The statistical significance of the identified terms and pathways was adjusted using a Q value with a stringent threshold (Q value ≤ 0.05).

### Western blotting

Western blotting was performed according to previously established protocols [41]. Lung tissue (50–100 mg) and BMDM were collected and homogenized in radioimmunoprecipitation assay (RIPA) lysis buffer (Cat No. AR0102-100, Boster, Wuhan, China) to extract proteins. Protein concentration was quantified using the bicinchoninic acid (BCA) protein assay kit (Cat No. AR0197, Boster, Wuhan, China), and the proteins from each sample were separated on SDS-PAGE gels (Cat No. PAGE10008, ygyr-biotech, Beijing, China) and transferred to PVDF membranes (Cat No. D032217, ygyr-biotech, Beijing, China). The membranes were subsequently blocked with a 5% milk solution (Cat No. P0216-300 g, Beyotime, Shanghai, China) in PBST buffer (Cat No. AR0194-10, Boster, Wuhan, China) and probed with diluted primary antibodies including anti- FGF2 (1:1000, Cat No. MA00121, Boster, Wuhan, China), BCL2(1:1000, Cat No.BM4985, Boster, Wuhan, China), BAX (1:1000, Cat No. BM3964, Boster, Wuhan, China ), β-actin (1:10000, Cat No. 66009-1-Ig, Proteintech, Wuhan, China), and GAPDH (1:10000, Cat No. BM1623, Boster, Wuhan, China) were used as primary antibodies.

### CLP model

Sepsis was induced using cecal ligation and puncture (CLP) surgery, as previously described [10]. Briefly, ten-week-old male mice were weighed and anesthetized via intraperitoneal injection of 1% pentobarbital at a dose of 40 mg/kg. An incision was made on the abdominal skin and muscles to expose the peritoneal cavity. The cecum was exteriorized and the distal portion was ligated using a 4 − 0 surgical suture. A 21G needle was used to create a through-and-through puncture (two holes) near the ligation site. The cecum was then carefully repositioned into the abdominal cavity and the mouse abdomen was sutured layer-by-layer with 4 − 0 surgical sutures. Mice in the sham group underwent cecal exposure without ligation or puncture. All mice received an immediate subcutaneous injection of 1 ml of preheated physiological saline at 37 °C for liquid resuscitation after surgery.

### Macrophage depletion and reconstitution

Macrophage depletion was performed as previously described [17]. Mice were injected with 0.2 ml of clodronate liposomes (Cat No. 40337ES, Yeasen, Shanghai, China) via the tail vein. After 48 h, the spleen cells were obtained by gently crushing the spleen. The cells were then labeled with anti-mouse MHCII-FITC (Cat No.107606, Biolegend, USA) and a rabbit polyclonal antibody F4/80 for 30 min before analysis using a flow cytometer (BD Cantoll) according to previously described methods [42]. Data were analyzed using the Diva software for FACS analysis. BMDM were isolated and cultured from WT and KO mice as previously described. Following the administration of chlorophosphonate liposomes, C57BL/6 mice were intravenously injected with 2 × 10^6^ BMDM two days later, and sepsis was subsequently induced by CLP 36 h after BMDM transplantation.

### Analysis of lung inflammation and lung vascular leak

Lung inflammation was assessed 24 h after sepsis induction using the following methods: (1) Measurement of BALF cell count. (2) Measurement of BALF cytokine and chemokine levels. The trachea was carefully incised to collect BALF by washing the lung tissue with precooled PBS. BALF cells were counted using a Countess II Automated Cell Counter. The levels of inflammatory cytokines TNFα, IL6, and IL1β were measured using an enzyme-linked immunosorbent assay (ELISA) kit. (3) Lung vascular leak was measured using the Evans blue assay (Cat No.MS4049, Maokang Bio, China). Briefly, the mice were administered 1% Evans blue dye solution in saline via tail vein injection. After 40 min, the mice were sacrificed and perfused via the heart, and lung tissues were collected. The lung weights were measured and placed in 1 ml of formamide (Cat No. 24390-14-5, AMRESCO, Shanghai, China) at 60 °C for 24 h to extract Evans blue dye. The concentration of Evans blue dye in the supernatant was quantified by measuring the absorbance at 620 nm and was calculated from a standard curve using a plate reader. (4) BALF protein concentrations. The total protein concentration in BALF was analyzed using the BCA method. (5) Lung wet-to-dry weight ratio. The lungs were harvested and weighed to determine the wet weight for each group. The wet lung was then dried in an oven at 60 °C for 72 h and re-weighed to determine the dry weight. This was calculated as the ratio of the wet weight to the dry weight.

### Lung histology and lung injury score

Twenty-four hours after CLP surgery, the lungs were fixed in 4% paraformaldehyde, embedded in paraffin, and stained with hematoxylin and eosin (HE). The lung injury index was determined using a previously described method. Briefly, lung injury was scored using semi-quantitative analysis, with a score of 0–4 based on pulmonary edema, alveolar and interstitial inflammation, alveolar and interstitial hemorrhage, atelectasis, and hyaline membrane formation. The scoring system assigned 0 points for no injury, 1 point for a lesion range of less than 25%, 2 points for a lesion range of 25–50%, 3 points for a lesion range of 50–70%, and 4 points for a full field of view. Each animal was evaluated by observing ten high-power fields and the average score was recorded.

### Statistical analysis

All results are shown as the mean ± standard deviation (mean ± SD) based on their distribution, and comparisons between the two groups were conducted using a two-tailed paired sample t-test. For analyses involving multiple groups, one-way analysis of variance was used to assess differences in data, followed by the Newman-Keuls multiple comparison test for correlation significance analysis. Statistical analyses were performed using GraphPad Prism 8.0, with the significance level set at *p* < 0.05.

## Supplementary Information

Supplementary Material 1.

Supplementary Material 2.

## Data Availability

All data produced or utilized in this study can be available from the corresponding author upon a reasonable request.
