# Supplementary material for: Deleting fibroblast growth factor 2 in macrophages aggravates septic acute lung injury by increasing M1 polarization and inflammatory cytokine secretion
Source: Mol Biomed. 2024 Oct 22;5:50. doi: 10.1186/s43556-024-00203-0 (PMC11496435; doi:10.1186/s43556-024-00203-0)
Supplement: Supplementary file 1 — Supplementary Material 1. [file 43556_2024_203_MOESM1_ESM.zip › 43556_2024_203_MOESM1_ESM/Supplementary Information-202409.docx]

**Deleting fibroblast growth factor 2 in macrophages aggravates septic acute lung injury by increasing M1 polarization and inflammatory cytokine secretion**

Lingxian Yi^#1,2^, Yu Chen^#3^, Yaoyang Zhang^3^, Haiquan Huang^3^, Jiahui Li^3^, Yirui Qu^2^, Tujun Weng^3*^, Jiake Chai^2*^

1 Chinese PLA Medical School, Department of Emergency, the Ninth Medical Centre of Chinese PLA General Hospital, Beijing, 100101, PR China

2 Chinese PLA Medical School, the Fourth Medical Centre of Chinese PLA General Hospital, Beijing, 100048, PR China;

3 Senior Department of Orthopaedics, the Fourth Medical Centre, Chinese PLA General Hospital, Beijing, 100048, PR China;

# These authors contributed equally to this work.

Corresponding authors: Tujun Weng, Email: wengtujun@163.com, [ORCID: 0000-0002-5417-7202](mailto:ORCID: 0000-0002-5417-7202 ) ; Jiake Chai, cjk304@126.com, ORCID: 0000-0003-1422-8594

**Abstract**

Septic lung injury is strongly associated with polarization of M1 macrophages and excessive cytokine release. Fibroblast growth factor (FGF) signaling plays a role in both processes. However, the impact of FGF2 deficiency on macrophage polarization and septic acute lung injury remains unclear. To investigate this, we obtained macrophages from FGF2 knockout mice and examined their polarization and inflammatory cytokine expression. We also eliminated endogenous macrophages using clodronate liposomes and administered FGF2 knockout or WT macrophages intravenously in conjunction with cecal ligation and puncture (CLP) surgery to induce sepsis. In vitro analysis by flow cytometry and real-time PCR analysis demonstrated that FGF2 deficiency resulted in increased expression of M1 markers (iNOS and CD86) and inflammatory cytokines (CXCL1, IL1β, and IL6), especially after LPS stimulation. Additionally, immunofluorescence demonstrated increased nuclear translocation of p65 NF-κB in FGF2 knockout macrophages and RNA-seq analysis showed enrichment of differentially expressed genes in the IL17 and TNFα inflammatory signaling pathways. Furthermore, in vivo experiments revealed that depletion of FGF2 in macrophages worsened sepsis-induced lung inflammation, lung vascular leak, and lung histological injury, accompanied by an increase in CD86-positive cells and apoptosis. Our study suggests that FGF2 deficiency in macrophages plays a critical role in the pathogenesis of septic ALI, possibly because of the enhanced M1 macrophage polarization and production of proinflammatory cytokines. These findings provide empirical evidence for potential therapeutic interventions targeting FGF2 signaling to modulate the polarization of M1 and M2 macrophages in the management of sepsis-induced acute lung injury.

**Keywords:** Fibroblast growth factor 2; M1 Macrophage; Sepsis; Acute lung injury; Inflammation

**Supplementary materials**

**1. ELISA measurement**

The BMDM cell supernatant and bronchoalveolar lavage fluid (BALF) were collected, and enzyme-linked immunosorbent assay (ELISA) was used to determine the levels of FGF2 (Cat No. ED-20227, LunChang Shuo Biotech, Shanghai, China), TNFα (Cat No. SEKM-0034, Servicebio, Beijing, China), IL1β(Cat No. SEKM-0002, Servicebio, Beijing, China), and IL6 (Cat No. SEKM-0007, Servicebio, Beijing, China). All ELISA experiments were performed in strict accordance with the manufacturer's instructions.

**2. In vivo fluorescence imaging**

In vivo fluorescence imaging of small animals was performed to assess the effectiveness of cell transplantation into mice (ABL X5, Tanon). Normal mice and those subjected to CLP were administered 2 × 10^6^ DIR(Cat No. KM0007, Biolabs, USA)-labeled BMDM via the tail vein. The small animal in vivo imaging system was employed at various time points post-injection to observe the survival and distribution of BMDM within living mice.

**3. Blood gas analysis**

Twenty-four hours after CLP, blood samples were collected from the abdominal aorta and analyzed using a blood gas detection test paper i-STAT CG4+Cartridge (Abbot Point of Care Inc. USA). Blood gas results were evaluated using a blood gas analyzer (Abbott i-STAT300G).

**4. TUNEL staining**

Twenty-four hours after CLP surgery, the lung tissues were embedded, sectioned, and subsequently analyzed for apoptotic cells using a TUNEL detection kit (Cat No. T6014S/T6014L, UElandy, China) following the manufacturer's instructions. Briefly, 50µl of TUNEL staining solution was applied to each tissue section and incubated at room temperature for 1h under light-proof conditions. Following incubation, the sections were washed with PBS, and the nuclei were counterstained with DAPI for 5 min. Apoptotic cells were assessed by observing at least ten randomly selected fields per section for TUNEL-positive staining.


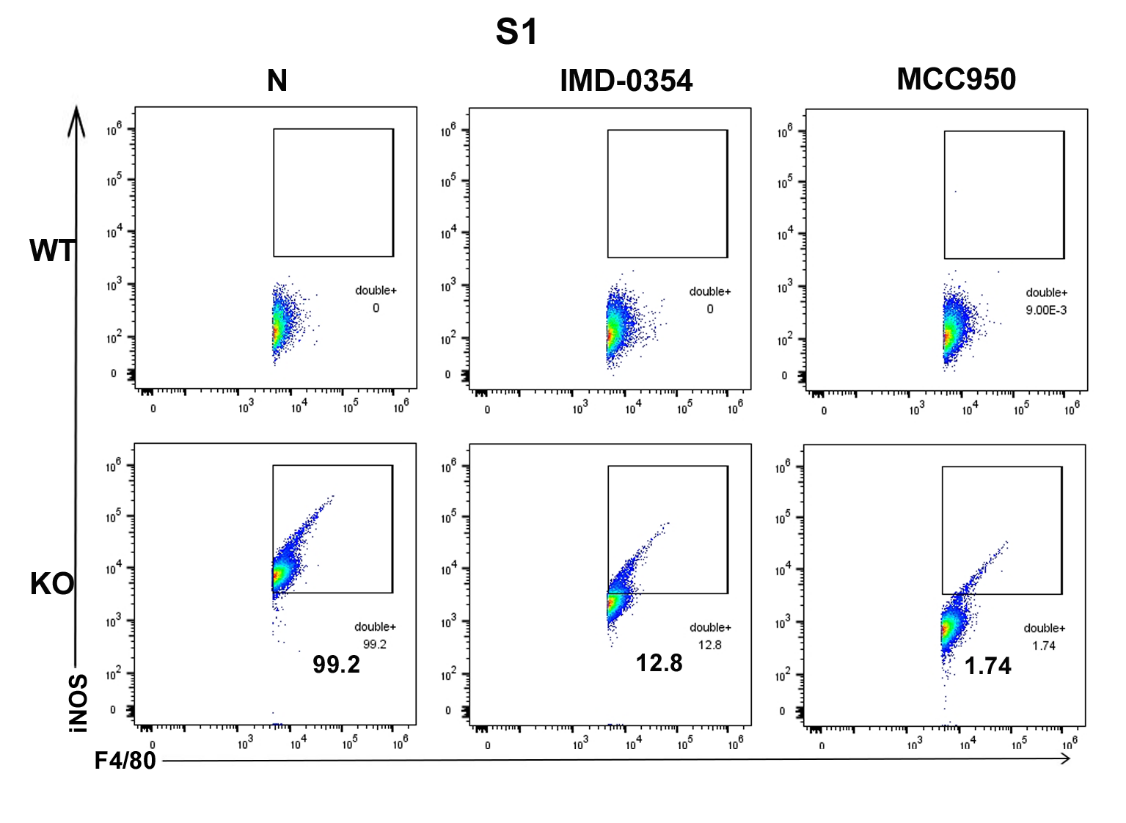


Figure S1. When FGF2 KO BMDM were treated with IMD0354, an inhibitor of NF-κB, or MCC950, a selective inhibitor of NLRP3 inflammasomes, a significant decrease in the percentage of iNOS-positive cells was observed by flow cytometry.


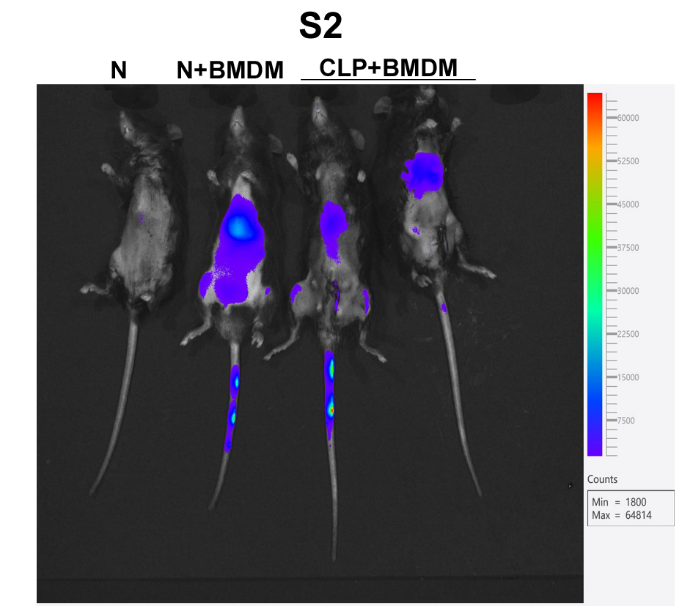


Figure S2. In vivo imaging results of DIR-labeled BMDM cells in mice three days after injection into the tail vein.


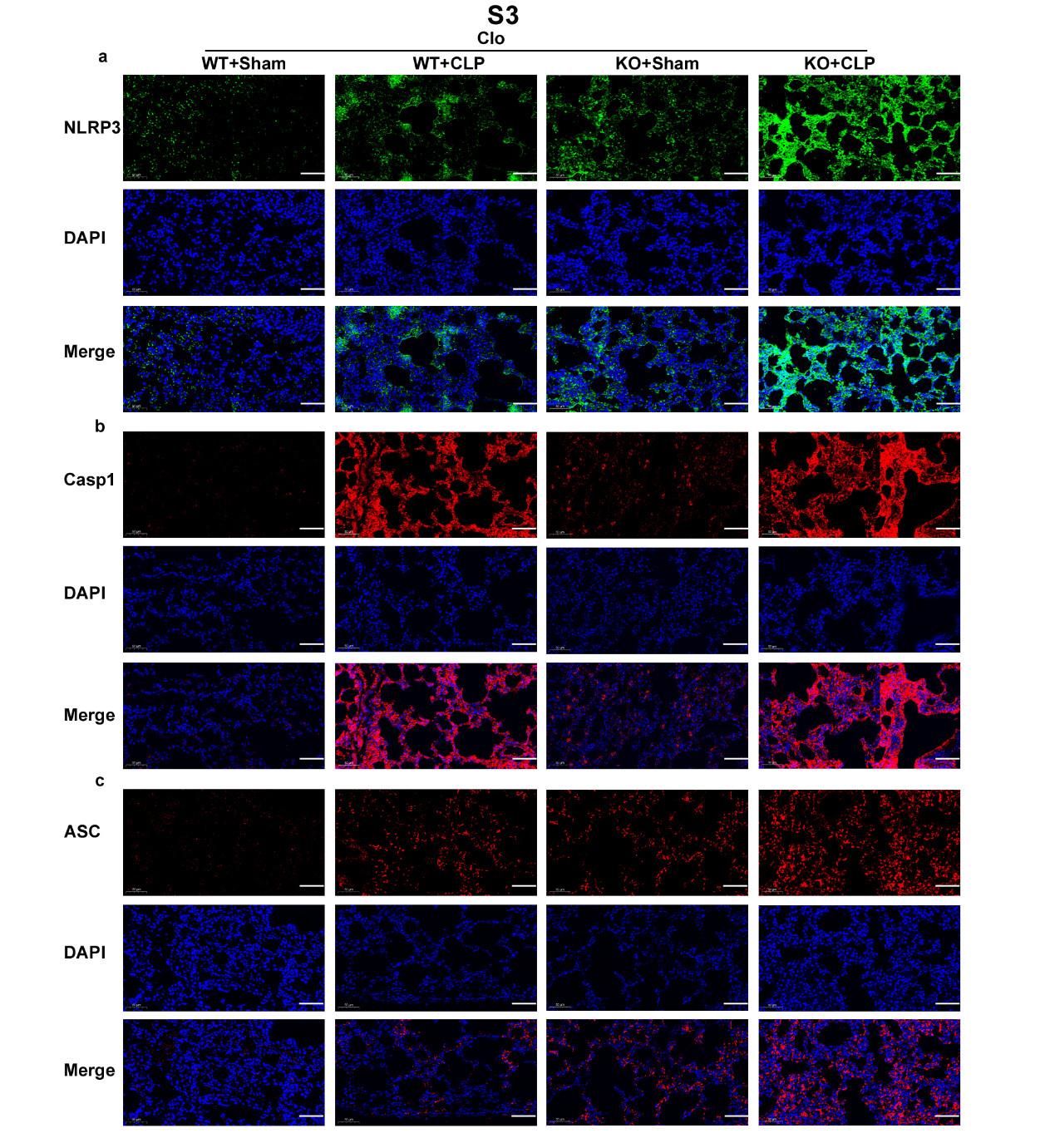


Figure S3. The aberrant expression of inflammasome-related markers, particularly NLRP3 (a), Caspase-1 p20 (b), and ASC (c), was observed in the lungs of the CLP group that was transplanted with FGF2 KO macrophages using immunofluorescence staining.

**Table 1. Primer sequences of RT-qPCR**

| Targets | Primer sequences |
| --- | --- |
| Cyclophilin A | F：CGAGCTCTGAGCACTGGAGA  R：TGGCGTGTAAAGTCACCACC |
| FGF2 | F：TTGTACACTCAAGGGGCTCTC  R：TTGAAGTGGCCTGGTGGGAA |
| Arg1 | F：AACGGGAGGGTAACCATAAGC  R：TGATGCCCCAGATGGTTTTC |
| CD206 | F：TTCGGTGGACTGTGGACGAGCA  R：ATAAGCCACCTGCCACTCCGGT |
| Fizz1 | F：TCCAGCTAACTATCCCTCCACTGT  R：GGCCCATCTGTTCATAGTCTTGA |
| MMP9 | F：GCGGCCCTCAAAGATGAACGG  R：GCTGACTACGATAAGGACGGCA |
| iNOS | F：CAGCTGGGCTGTACAAACCTTT  R：CATTGGAAGTGAAGCGTTTCG |
| IL6 | F：CCAGTTGCCTTCTTGGGACT  R：GGTCTGTTGGGAGTGGTATCC |
| IL10 | F：AAGCTCCAAGACCAAGGTGTC  R：CACACTGCAGGTGTTTTAGCTT |
| CXCL1 | F：TTGACGCTTCCCTTGGACAT  R：CTTTGAACGTCTCTGTCCCGA |
| IL1β | F：CCCTGAACTCAACTGTGAAATAGCA  R：CCCAAGTCAAGGGCTTGGAA |
| TNFα | F：ATACACTGGCCCGAGGGAAC  R：CCACATCTCGGATCATGCTTTC |
| IL18 | F：GACAGCCTGTGTTCGAGGAT  R：GGTGGATCCATTTCCTCAAAGG |
| YM1 | F：TGGAATTGGTGCCCCTACAA  R：CCACGGCACCTCCTAAATTG |
