# Supplementary figures and images for: Deleting fibroblast growth factor 2 in macrophages aggravates septic acute lung injury by increasing M1 polarization and inflammatory cytokine secretion
Source: Mol Biomed. 2024 Oct 22;5:50. doi: 10.1186/s43556-024-00203-0 (PMC11496435; doi:10.1186/s43556-024-00203-0)

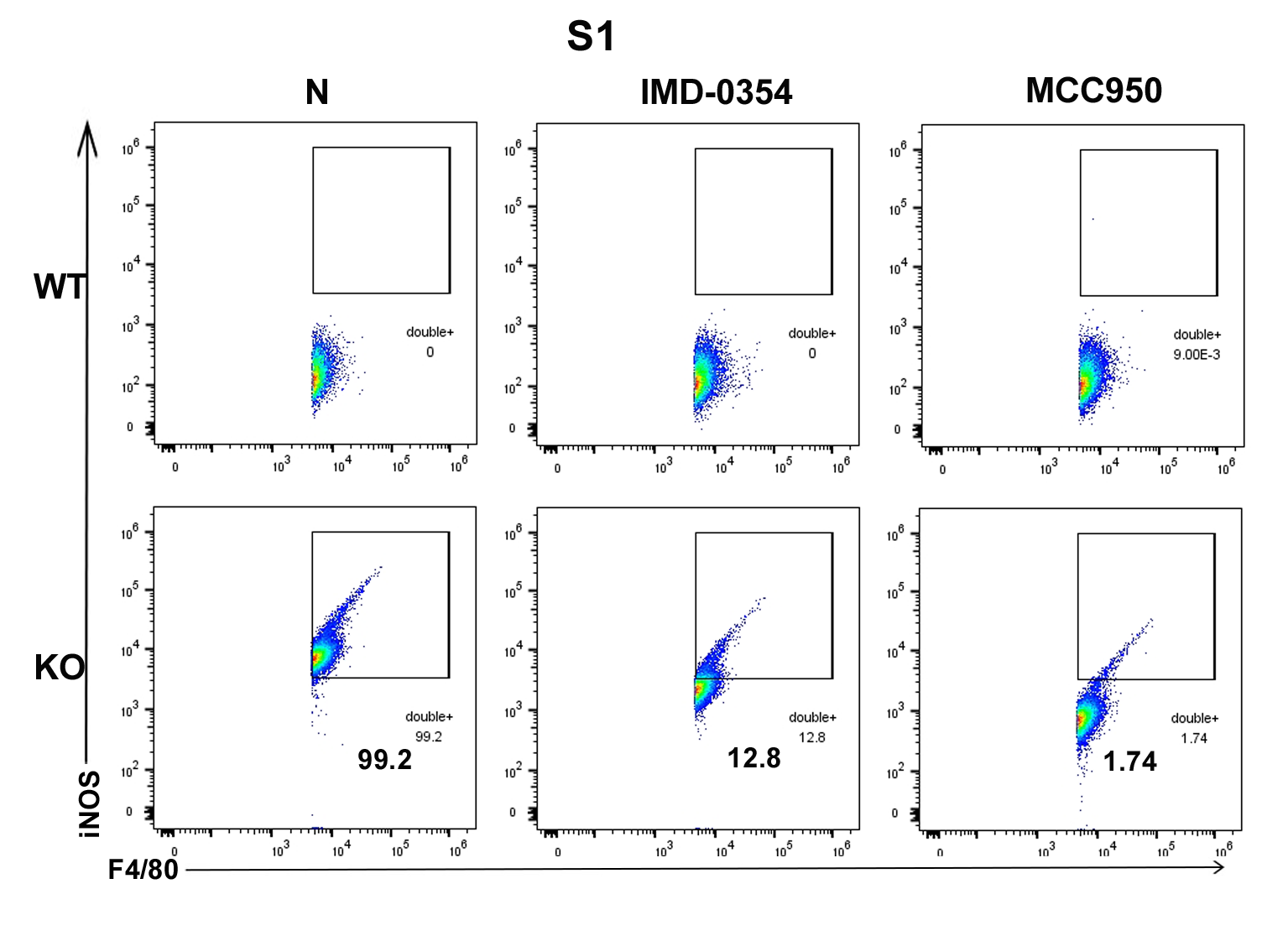

Supplement: Supplementary file 1 — Supplementary Material 1. [file 43556_2024_203_MOESM1_ESM.zip › 43556_2024_203_MOESM1_ESM/S1.tif]

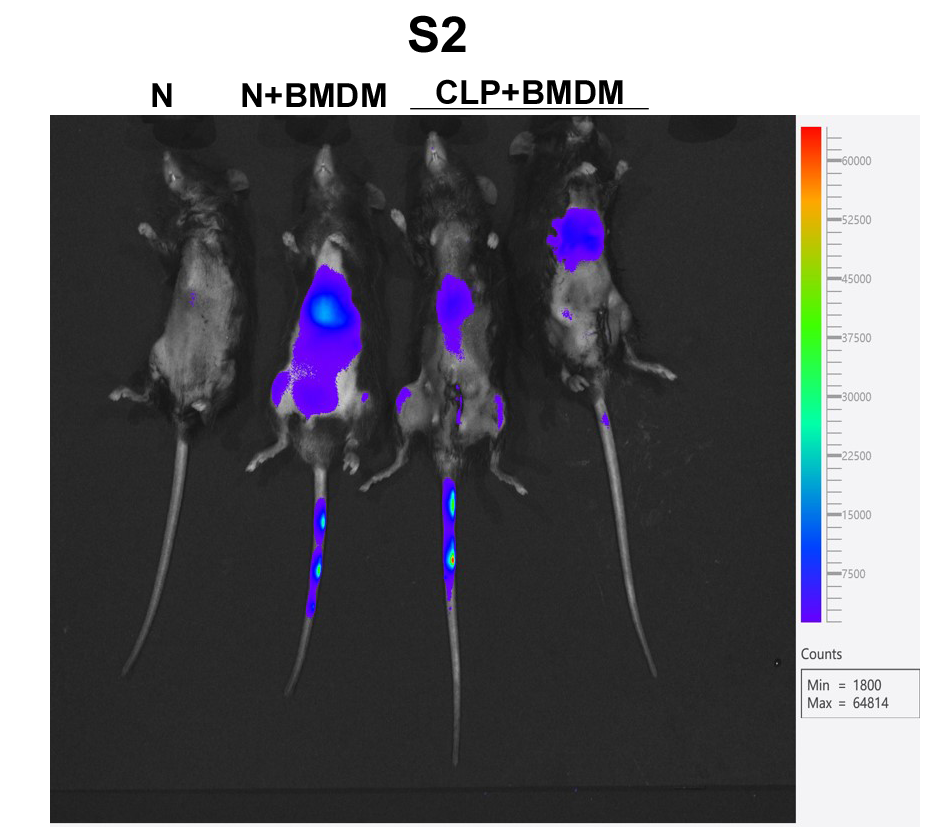

Supplement: Supplementary file 1 — Supplementary Material 1. [file 43556_2024_203_MOESM1_ESM.zip › 43556_2024_203_MOESM1_ESM/S2.tif]

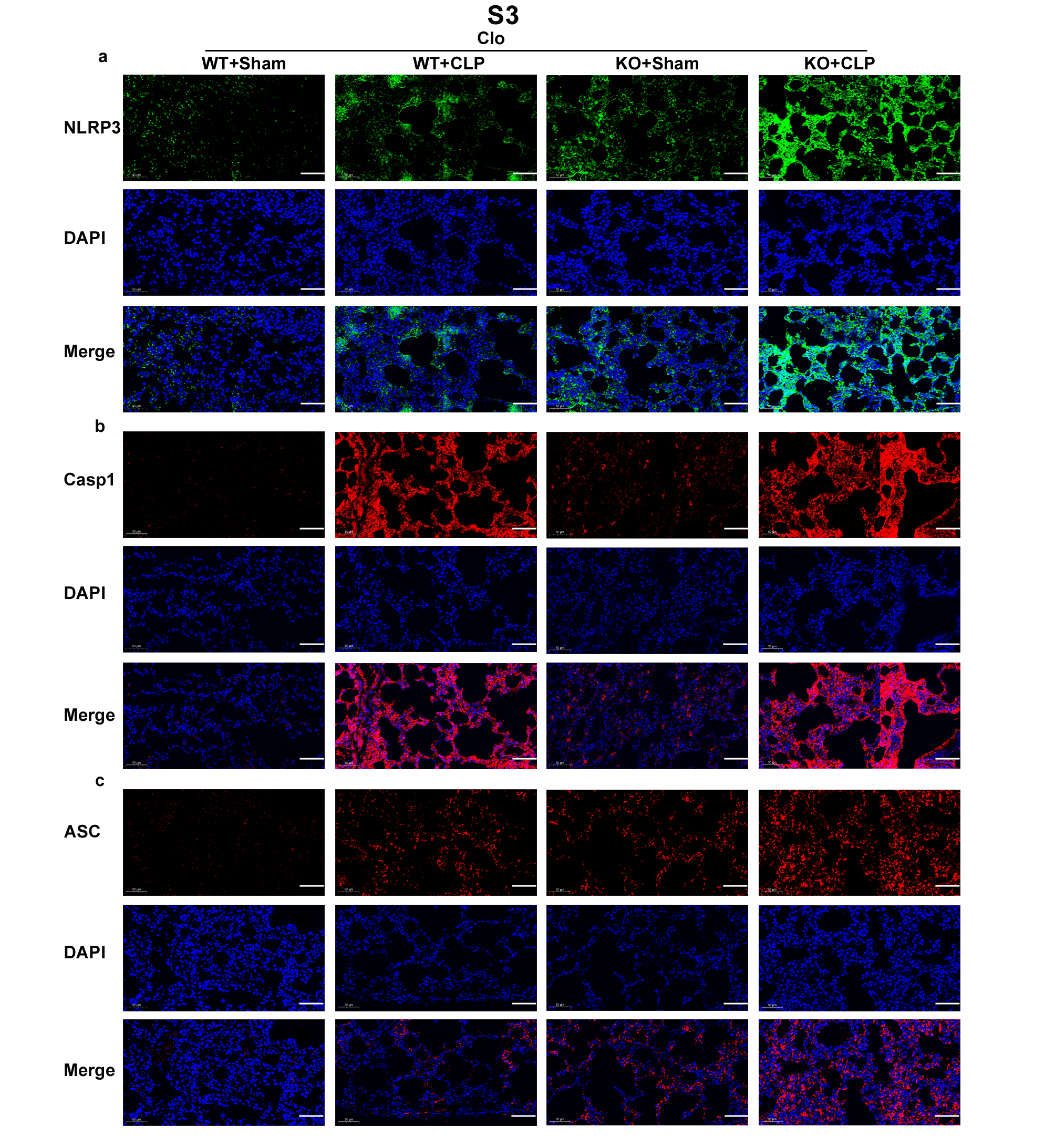

Supplement: Supplementary file 1 — Supplementary Material 1. [file 43556_2024_203_MOESM1_ESM.zip › 43556_2024_203_MOESM1_ESM/S3.tif]
