# Supplementary material for: Deleting fibroblast growth factor 2 in macrophages aggravates septic acute lung injury by increasing M1 polarization and inflammatory cytokine secretion
Source: Mol Biomed. 2024 Oct 22;5:50. doi: 10.1186/s43556-024-00203-0 (PMC11496435; doi:10.1186/s43556-024-00203-0)

The whole-membrane results of BCL2 and GAPDH is listed as below:


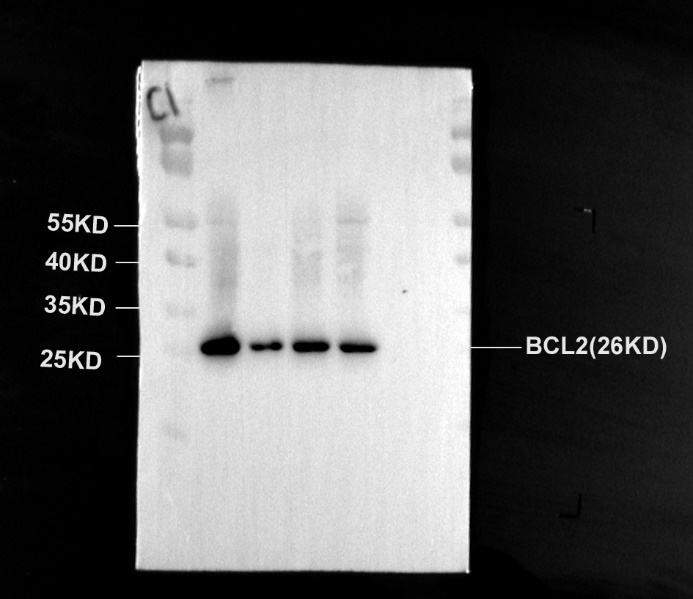




The whole-membrane results of BAX and GAPDH is listed as below:


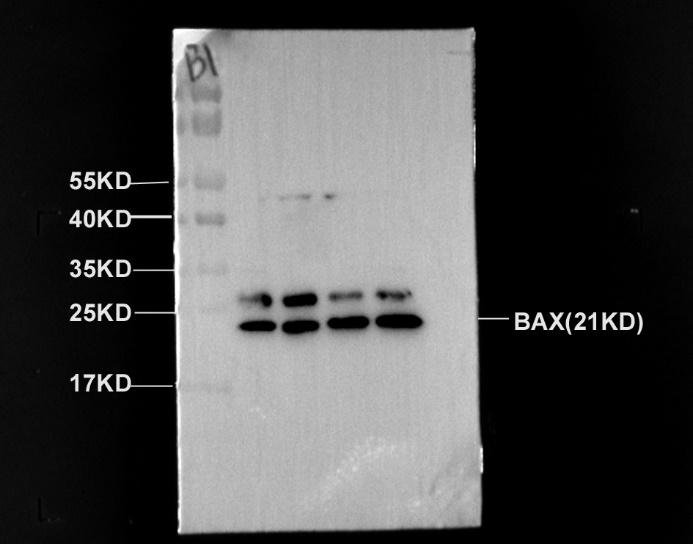

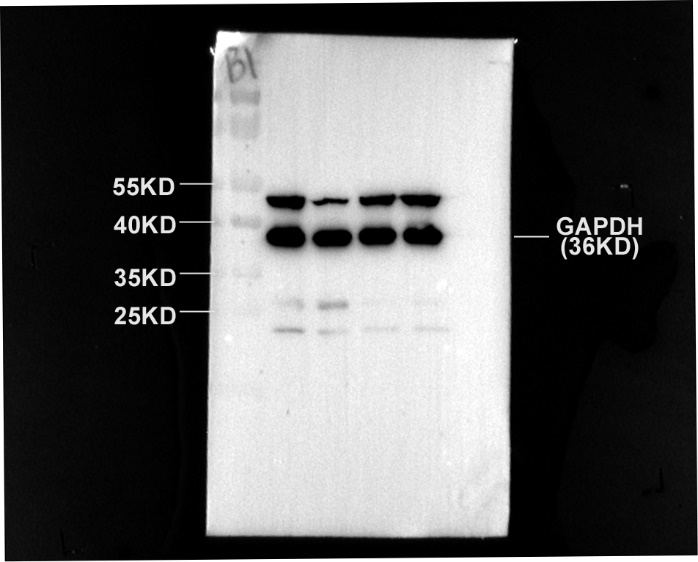

Supplement: Supplementary file 2 — Supplementary Material 2. [file 43556_2024_203_MOESM2_ESM.docx]
